# The Effect of Magnesium Intake on Stroke Incidence: A Systematic Review and Meta-Analysis With Trial Sequential Analysis

**DOI:** 10.3389/fneur.2019.00852

**Published:** 2019-08-07

**Authors:** Binghao Zhao, Lei Hu, Yifei Dong, Jingsong Xu, Yiping Wei, Dongliang Yu, Jianjun Xu, Wenxiong Zhang

**Affiliations:** ^1^Department of Thoracic Surgery, The First Affiliated Hospital of Nanchang University, Nanchang, China; ^2^Departments of Neurosurgery, Peking Union Medical College, Peking Union Medical College Hospital, Chinese Academy of Medical Sciences, Beijing, China; ^3^Department of Neurology, The Second Affiliated Hospital of Nanchang University, Nanchang, China; ^4^Department of Cardiology, The Second Affiliated Hospital of Nanchang University, Nanchang, China

**Keywords:** magnesium, stroke, meta-analysis, trial sequential analysis, systematic review

## Abstract

**Background:** The effect of magnesium on stroke has been consistently discussed less, and the results of previous studies have been contradictory. We reviewed the latest literature and quantified robust evidence of the association between magnesium intake and stroke risk.

**Methods:** PubMed, EMBASE, the Cochrane Library, the Web of Science and ClinicalTrials.gov were searched through inception to January 15, 2019 for prospective cohort studies on magnesium intake and the incidence of stroke.

**Results:** Fifteen studies with low bias involving 18 cohorts were entered into this study. The summary relative risk (RR) was significantly reduced by 11% for total stroke (RR: 0.89 [95% CI, 0.83–0.94]; *P* < 0.001) and by 12% for ischemic stroke (RR: 0.88 [95% CI, 0.81–0.95]; *P* = 0.001), comparing the highest magnesium intake category to the lowest. After adjusting for calcium intake, the inverse association still existed for total stroke (RR: 0.89 ([95% CI, 0.80–0.99]; *P* = 0.040). There was an inverse but non-significant association for hemorrhagic stroke, subarachnoid hemorrhage and intracerebral hemorrhage. The quantitative associations for total and ischemic stroke were robust. Importantly, high-risk females who had a body mass index (BMI) ≥25 kg/m^2^ and who were subjected to a ≥12 y follow-up exhibited a greater decrease in RRs as a result of magnesium intake. For each 100 mg/day increase in magnesium, the risk for total stroke was reduced by 2% and the risk for ischemic stroke was reduced by 2%.

**Conclusions:** Increasing magnesium intake may be a crucial component of stroke prevention that acts in a dose-dependent manner. However, the conclusion is limited by the observational nature of the studies examined, and further randomized controlled trials are still needed.

## Key Points

1. We conducted a quantitative analysis that suggested that magnesium intake has a strong inverse association with total stroke.
2. Magnesium-rich food consumption should be recommended in dietary guidelines for high-risk individuals.
3. Highlighting early management or intervention of stroke requires various efforts and strategies.
4. This study, which includes a considerable amount of evidence, assists with innovation of the global dietary pattern.
5. This can be regarded as the latest meta-analysis for the association between magnesium intake and total stroke and ischemic stroke.

## Introduction

Magnesium acts as a critical cofactor for hundreds of enzymes involved in glucose metabolism, protein production, and nucleic acid synthesis (1, 2). An intake deficiency has been reported to be associated with many diseases, such as Alzheimer's disease, asthma, attention deficit hyperactivity disorder, type 2 diabetes, hypertension, cardiovascular disease (e.g., stroke), migraine headaches, osteoporosis, and cancer (1, 2).

The American Heart Association (AHA)/American Stroke Association (ASA) (3) guidelines suggest following a Mediterranean diet to reduce sodium intake and increase potassium consumption. Despite the dietary pattern guidelines, the recommendations regarding other special nutrients, such as magnesium, have been limited, and most of the current investigations are concentrated on sodium, potassium or calcium. In past years, studies (1, 2, 4–16) of magnesium have emerged; however, the data on the topic are limited due to the challenge of conducting long-term follow-ups. Previous meta-analyses (14, 17–19) supported a mild benefit of magnesium intake for stroke patients, but these findings are insufficient to make specific recommendations and are not conclusive for dose-response patterns. We performed a more comprehensive meta-analysis with state-of-the-art statistical methods and new evidence (1) to estimate the effect of magnesium intake on stroke and to update the current evidence; (2) to apply trial sequential analysis (TSA) to determine whether current observational studies are conclusive; and (3) to establish a detailed dose-response relationship.

## Methods

This study was performed according to the Cochrane Handbook and the Preferred Reporting Items for Systematic Reviews and Meta-Analyses (PRISMA) guidelines (Table S1) (Registration information: PROSPERO CRD42018099363).

### Search Strategy

PubMed, EMBASE, Cochrane Library, Web of Science, and ClinicalTrials.gov were rigorously reviewed through inception to January 15, 2019 for studies on magnesium intake and stroke, without language restrictions. We used the following key words: “magnesium,” “Stroke,” “Cerebrovascular Stroke,” “Cohort Studies,” and “Prospective Studies.” The electronic searches were complemented by a manual search of the reference lists of retrieved studies.

### Selection Criteria

We chose eligible studies according to the “PICOS” principles, and eligible studies had to meet the following criteria:

1. Population: individuals with certain dietary/energy intake who had no current stroke. Their current life expectancy was sufficient for proper follow-up, and their essential data were available;
2. Exposure: magnesium, including dietary and total intake (dietary and supplementary magnesium);
3. Outcome: total stroke, ischemic stroke, and hemorrhagic stroke [including subarachnoid hemorrhage and intracerebral hemorrhage according to anatomical site or presumed etiology (20)]; and
4. Study design: prospective cohort studies.

The follow-up period of the included studies was no <1 y. We excluded studies involving populations with prevalent cancer, stroke at baseline, implausibly low or high energy intake, or missing information; reviews; basic studies; meta-analyses; etc.

### Data Extraction and Quality Assessments

Two investigators (YW and JX) independently extracted the following information: the first author, publication year, period of cohort studies, duration of persistent exposure, basic characteristics of the enrolled participants (age, region, BMI, etc.), median magnesium intake for each quantile (tertile, quartile, or quintile), total stroke cases, subtypes of total stroke, dietary and cases assessments, and adjusted confounding covariates. The adjusted relative risk (RR) and hazard ratio (HR) of the main outcomes in fully adjusted models were specifically extracted. Discrepancies were resolved by a discussion with a third author (WZ).

Three authors (BZ, WZ, and DY) assessed study methodological quality using the Newcastle-Ottawa Scale (NOS) (21). On a 0–10 scale, each study was categorized as low (0–5), medium (6–7), or high (8–10) quality.

### Statistical Analysis

Publications reporting data separately for men and women or based on different types of diseases were treated as independent cohorts. Multivariate RRs and the corresponding 95% confidence intervals (95% CIs) for stroke risk for the highest category vs. the lowest category of magnesium intake and other outcomes were estimated with the DerSimonian-Laird (D-L) random effects model because the assumptions involved accounted for the presence of within-study and between-study variability. The adjusted HRs in the primary studies were considered approximate RRs. Fully adjusted effect sizes (ESs) were logarithmically transformed to stabilized variance, and the distribution was normalized.

Between-study heterogeneity was determined with the Cochran Q chi-square test and *I*^2^. An *I*^2^> 50% or a *P*-value for the Q test <0.1 was regarded as indicating significant heterogeneity. In addition, a sensitivity analysis was performed by omitting one study each time to obtain an understanding of the causes of the heterogeneity. We conducted post-subgroup analyses to determine the influence of other clinical factors (e.g., participant region, sex, mean BMI, etc.).

Publication bias was investigated by Egger's linear regression tests with *P* < 0.10 indicating significant bias. All analyses were performed using Stata version 14.0 (StataCorp, College Station, TX, USA); two-sided *P* < 0.05 was statistically significant, except where otherwise specified.

### Trial Sequential Analysis

Random errors stem from sparse data, and repetitive testing of accumulating data increases the risk of type 1 error (false positive). The risk of type 1 error can be reduced by TSA with TSA software (version 0.9 beta, http://www.ctu.dk/tools-and-links) because this analysis combined the estimation of required information size (RIS) with an adjusted threshold for statistical significance (22). This method could reveal whether the evidence was abundant and conclusive in our meta-analysis. If the Z-curve crosses the TSA boundary or enters the futility area, a sufficient effect is reached, and further studies are not needed; if not, the evidence in our study would be insufficient. The TSA was performed for a 5% relative risk reduction for stroke outcome, a 10% reduction for subarachnoid and intracerebral hemorrhage outcome, and conservatively, according to the TSA manual, a 5% (α = 0.05; two-sided) total risk of type 1 error and 80% statistical power.

### Dose-Response Analysis

The methods used for the dose-response analyses of the main outcomes were proposed by Orsini et al. (23). The categories of magnesium intake, distributions of cases and person-years, RR and 95% CI were extracted. Once the number of cases and/or person-years was not available, variance-weighted least squares regression was used to pool the risk estimate. For most studies, the median intake for each quantile (tertile, quartile or quintile) of magnesium intake was assigned as the representative dose. For continuous intake reported as categorical data with a range in some studies, we assigned the midpoint category of the lower and upper bounds to the RR. When the highest category was open-ended, we assumed the range of the open-ended interval to be 1.5 times that of the adjacent interval; when the lowest category was open, we assumed the range the open-ended interval to be 1.5 times that of the range of the adjacent interval in the category. We determined generalized least squares regression models to calculate study-specific RR estimates per 50, 100, and 150 mg/day increase in magnesium intake if there was evidence for linear relationships. In addition, the non-linear relationships between magnesium intake and all types of stroke were evaluated using restricted cubic splines with four knots located at the 5th, 35th, 65th, and 95th percentiles of the distribution. The *P*-value for curve linearity or nonlinearity was calculated by testing the null hypothesis that the coefficient of the second spline is equal to zero.

All results were presented using two-stage dose-response model plots (including linear and non-linear relationships). The results of <50 mg/day, ≥50 and <100 mg/day, ≥100 and <150 mg/day, and ≥150 mg/day increments were displayed in forest plots.

## Results

### Study Selection and Characteristics

Of 5,037 studies (4,948 from the mentioned databases and 89 from other available literature), 4,973 studies were excluded during the initial screening, and 49 studies were excluded after full consideration (Figure S1).

The 15 (4–8) publications, involving 18 cohorts with 692,998 participants and 20,138 total stroke cases, were entered into our analysis (mean follow-up, 14.4 y; mean BMI, 24.9 kg/m^2^; most middle-aged). Regarding the subtypes of total stroke, 15 reports from 12 publications (1, 2, 5–11, 13, 14, 16) revealed the associations with ischemic stroke, and 10 cohorts from 8 publications (2, 5–7, 9, 13, 14, 16) revealed hemorrhagic stroke results. Of those eligible, 6 studies (1, 4–6, 13, 14) were conducted in North America (America), 5 (2, 7, 11, 12, 15) in Europe **(**Sweden, the Netherlands and the United Kingdom), and 4 (8–10, 16) in Asia (Taipei and Japan). Only male patients were considered in 3 studies (4, 7, 13), and only females were considered in 4 studies (2, 5, 6, 14); the other 8 studies (1, 8–12, 15, 16) enrolled male and female patients. Most of the eligible studies used the food frequency questionnaire (FFQ) or the semiquantitative FFQ (SFFQ) to assess each individual's diet. Seven studies (1, 6, 9–11, 15, 16) used dietary magnesium intake, and 8 studies (2, 4, 5, 7, 8, 12, 14) used total magnesium intake. Of note, the adjusted confounders were mostly alike; however, the adjusted dietary confounders, such as cereal fiber, potassium, and calcium, varied across the individual studies (no studies adjusted for sodium). All papers were written in English (Table S2). The average NOS score was 8.93; thus, all included studies were of high quality. Among all studies, the study by Sluijs et al. (11) was the only one to provide no assessment of outcomes (Table S3).

### Synthesis of Total Stroke, Ischemic Stroke, and Hemorrhagic Stroke

**Figure 1:**
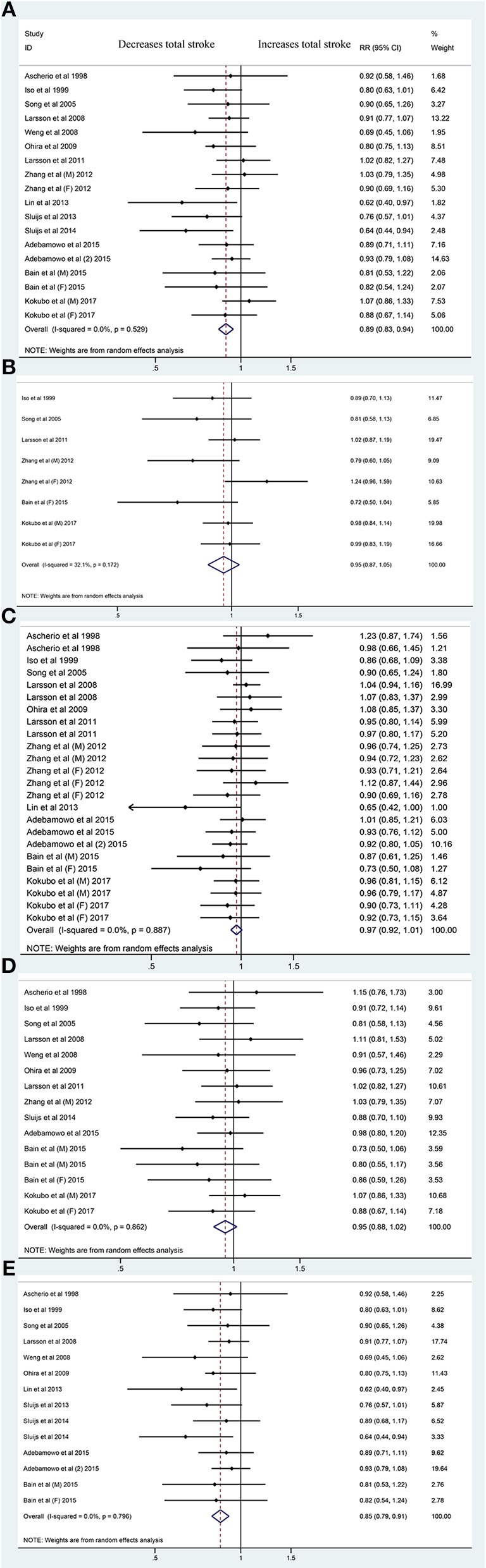
Forest plots of the risk of total stroke for magnesium intake **(A)** and for <50 mg/day **(B)**, ≥50 and <100 mg/day **(C)**, ≥100 and <150 mg/day **(D)**, and ≥150 mg/day Magnesium Increments **(E)**. A total of 15 publications including 18 cohorts: reporting data separately for males and females (9, 15, 16) within an article were treated as independent studies. RR, relative risk.

**Figure 2:**
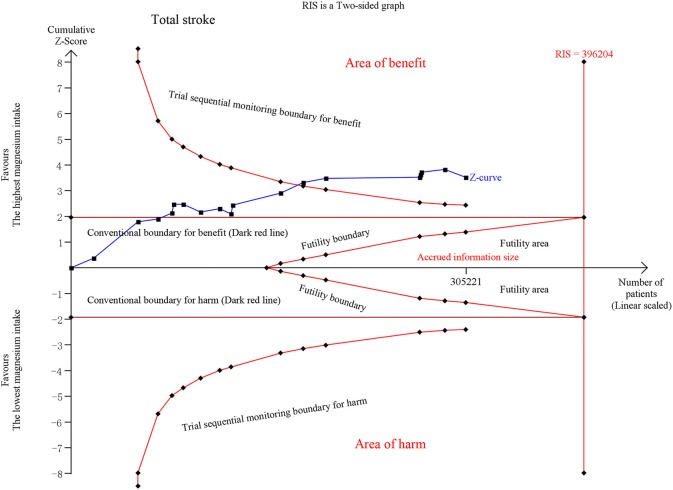
Trial Sequential Analysis (TSA) of total stroke comparing the highest magnesium intake category to the lowest. The TSA illustrated that the cumulative Z-curve crossed both the conventional boundary for benefit and the trial sequential monitoring boundary for benefit, demonstrating that the results are robust and conclusive, and further studies are not required. A diversity required information size (RIS) of 396,204 was computed by α = 0.05 (two-sided); 80% statistical power, with a conservative relative risk reduction of 5%. X-axis, the number of patients; Y-axis, cumulative *Z*-score; Dark red lines, conventional boundaries (upper for benefit, *Z*-score = 1.96; lower for harm, *Z*-score = −1.96; two-sided *P* = 0.05); Sloping red lines with black, filled circle icons, trial sequential monitoring boundaries (two sides, computed accordingly); Sloping blue line with black, filled circle icons, Z-curve; Vertical red full line, RIS computed accordingly; Upper conventional boundary for benefit, area of benefit; Lower conventional boundary for harm, area of harm; Middle area, futility area; Red lines with black, filled circle icons in the futility area, futility boundaries.

Fifteen publications on total stroke showed that the RR was reduced by 11% (RR, 0.89 [95% CI, 0.83–0.94]; *P* < 0.001) with no heterogeneity (*I*^2^ = 0%; *P* = 0.529) in the highest category vs. the lowest category. For the dose-category-specific analyses, a trend of reduced RR was found in <50 mg/day increments (RR, 0.95 [95% CI, 0.87–1.05]; *P* = 0.331), ≥50 and <100 mg/day (RR, 0.97 [95% CI, 0.92–1.01]; *P* = 0.108), and ≥100 and <150 mg/day (RR, 0.95 [95% CI, 0.88–1.02]; *P* = 0.154), but the results were not significant. Regarding the ≥150 mg/day increments, the risk decreased by 15% (RR, 0.85 [95% CI, 0.79–0.91]; *P* < 0.001) (Figure 1). Although the pooled ESs did not exceed the RIS, the TSA established sufficient and conclusive evidence. Therefore, further observational studies are not required and are less likely to affect the conclusion (Figure 2, Figure S2).

**Figure 3:**
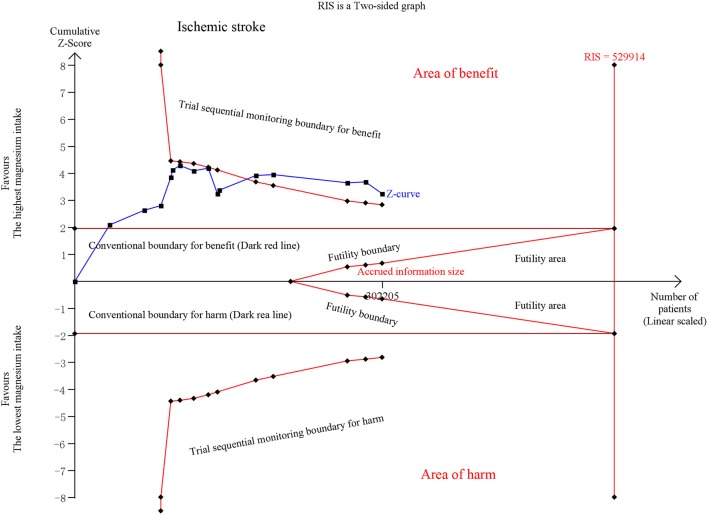
Trial sequential analysis of ischemic stroke comparing the highest magnesium intake category to the lowest.

Iso et al. (5) provided results for ischemic stroke and ischemic stroke excluding non-atherogenic stroke. Thirteen publications on ischemic stroke revealed that the estimated RR decreased by 12% (RR, 0.88 [95% CI, 0.81–0.95]; *P* = 0.001) with no significant heterogeneity (*I*^2^ = 16.9%; *P* = 0.265). No significant association was observed in <50 mg/day increments (RR, 0.94 [95% CI, 0.85–1.04]; *P* = 0.232), ≥50 and <100 mg/day (RR, 0.98 [95% CI, 0.93–1.03]; *P* = 0.419), or ≥100 and <150 mg/day (RR, 0.95 [95% CI, 0.87–1.04]; *P* = 0.249). The risk was reduced by 16% in the ≥150 mg/day increment analysis (RR, 0.84 [95% CI, 0.78–0.91]; *P* < 0.001) (Figure S3). TSA exhibited conclusive results and revealed that the relationship could hardly be altered by further trials (Figure 3, Figure S4).

Eight studies found that the risk of hemorrhagic stroke was not significantly associated with magnesium intake (RR, 0.93 [95% CI, 0.82–1.06]; *P* = 0.282) nor were the <50 mg/day (RR, 1.00 [95% CI, 0.87–1.15]; *P* = 0.991), ≥50 and <100 mg/day (RR, 0.95 [95% CI, 0.85–1.07]; *P* = 0.405), ≥100 and <150 mg/day (RR, 0.91 [95% CI, 0.75–1.11]; *P* = 0.374), and ≥150 mg/day magnesium increments (RR, 0.96 [95% CI, 0.80–1.15]; *P* = 0.681) (Figure S5). The results on hemorrhagic stroke are not conclusive, and we still require further studies (Figures S6, S7).

Three (2, 5, 7) studies disclosed that the RR of subarachnoid hemorrhage was not significantly reduced (RR, 0.99 [95% CI, 0.71–1.39]; *P* = 0.963). A non-significantly reduced RR was also detected in the <50 mg/day (*P* = 0.108), ≥50 and <100 mg/day (*P* = 0.521), ≥100 and <150 mg/day (*P* = 0.330), and ≥150 mg/day (*P* = 0.630) increments (Figure S8). TSA suggested conclusive and robust results (Figure S9).

Three (2, 5, 7) studies restricted to intracerebral hemorrhage revealed a non-significantly reduced RR (RR, 0.92 [95% CI, 0.71–1.20]; *P* = 0.540). The dose-category-specific analyses revealed no significant association for the <50 mg/day (*P* = 0.108), ≥50 and <100 mg/day (*P* = 0.974), ≥100 and <150 mg/day (*P* = 0.767), or ≥150 mg/day (*P* = 0.461) increments (Figure S10). Additionally, further studies are still required to draw robust conclusions (Figure S11).

### Sensitivity and Subgroup Analyses

A sensitivity analysis conducted by omitting one study each time was not conducted because no heterogeneity was found. Only one study (2) on total stroke was adjusted for cereal fiber intake. After potassium intake adjustment, the RR was 0.92 [[95% CI, 0.82–1.02]; *P* = 0.097; *n* = 5 (10, 20–22, 24)] and the RR was 0.89 [[95% CI, 0.80–0.99]; *P* = 0.040; *n* = 5 (11, 20–22, 24)] after calcium intake adjustment. Overall, magnesium was still shown to have a mild inverse association with total stroke after adjusting for calcium intake.

In the comprehensive stratified analysis, most of the results regarding total stroke and ischemic stroke remained significant across the subgroups. Notably, individuals in North America and Europe received greater benefits than those in Asia. Magnesium intake was also shown to offer advantages to obese participants (mean BMI ≥ 25 kg/m^2^) (RR, 0.89 [95% CI, 0.82–0.96] for total stroke; 0.88 [95% CI, 0.81–0.96] for ischemic stroke) whose follow-ups were longer (≥12 y) (RR, 0.89 [95% CI, 0.83–0.95] for total stroke; 0.88 [95% CI, 0.81–0.95] for ischemic stroke). Total and dietary magnesium intake were both associated with a significantly reduced risk of total stroke; however, total magnesium intake showed greater effects than dietary intake on ischemic stroke (RR, 0.87 [95% CI, 0.80–0.94]). Likewise, a ≥180 mg/day difference between the highest and the lowest intakes had greater benefits for ischemic stroke (RR, 0.83 [95% CI, 0.76–0.91]) than <180 mg/day. Interestingly, the female-specific RR for total stroke was significantly decreased by magnesium intake (RR, 0.91 [95% CI, 0.83–0.99], *P*_interation_ = 0.031) and decreased relatively for ischemic stroke (RR, 0.89 [95% CI, 0.79–1.00]) compared with male RR. Although CV events (excluding stroke), hypercholesterolemia and diabetes were all risk factors for stroke, magnesium intake could still exhibit a trend of an inverse association with total and ischemic stroke in individuals, even with those risk factors. We did not observe a significantly reduced risk of hemorrhagic stroke in the subgroup analyses. These results are outlined in Table 1.

**Table 1:** Subgroup analyses relating to magnesium intake and total stroke, ischemic stroke, hemorrhagic stroke.

**Group**	**Total stroke**				**Ischemic stroke**				**Hemorrhagic stroke**			
	**No. of studies**	**RR (95% CI)**	***I^**2**^* (%)**	***P_***interation***_***	**No. of studies**	**RR (95% CI)**	***I^**2**^* (%)**	***P_***interation***_***	**No. of studies**	**RR (95% CI)**	***I^**2**^* (%)**	***P_***interation***_***
Total	15	0.89 (0.83–0.94)	0		12	0.88 (0.81–0.95)	16.9		8	0.93 (0.82–1.06)	46.1	
**Participants region**												
Total	15			0.733	12			0.584	8			0.873
North America	6	0.87 (0.79–0.96)	0		5	0.85 (0.76–0.95)	0		4	0.90 (0.71–1.15)	0	
Europe	5	0.87 (0.77–0.98)	14.8		3	0.86 (0.78–0.95)	0		2	0.99 (0.79–1.25)	0	
Asia	4	0.90 (0.78–1.05)	32.8		4	0.93 (0.75–1.14)	45.5		2	0.89 (0.66–1.21)	53.4	
Multiple nations	0	NA	NA		0	NA	NA		0	NA	NA	
**Sex^a^**												
Total	18			0.031	14			0.134	10			0.425
Male	6	0.95(0.86–1.05)	0		4	0.99 (0.82–1.19)	52.8		4	0.97 (0.75–1.26)	35.5	
Female	7	0.91 (0.83–0.99)	0		6	0.89 (0.79–1.00)	0		6	0.88 (0.74–1.06)	0	
Both^b^	5	0.74 (0.64–0.85)	0		4	0.76 (0.65–0.88)	0		0	NA	NA	
**Mean BMI (kg/m^2^**)****												
Total	15			0.606	12			0.631	8			0.418
≥ 25	8	0.89 (0.82–0.96)	0		6	0.88 (0.81–0.96)	0		5	0.97 (0.81–1.17)	0	
< 25	5	0.89 (0.78–1.01)	30		5	0.87 (0.73–1.03)	44		3	0.88 (0.69–1.12)	39.3	
Unknown	2	0.80 (0.63–1.02)	0		1	0.76 (0.57–1.07)	NA		0	NA	NA	
**Follow–up duration (y)**												
Total	15			0.798	12			0.811	8			0.808
≥ 12	11	0.88 (0.82–0.94)	5.3		10	0.87 (0.80–0.95)	19.1		7	0.93 (0.81–1.08)	7.7	
< 12	4	0.90 (0.77–1.05)	0		2	0.86 (0.62–1.20)	48.4		1	0.88 (0.57–1.36)	NA	
**Dietary assessment**												
Total	15			0.578	12			NA	8			NA
FFQ/validated FFQ	14	0.89 (0.83–0.95)	3.8		12	0.88 (0.81–0.95)	16.9		8	0.93 (0.82–1.06)	0	
SFFQ/validated SFFQ	0	NA	NA		0	NA	NA		0	NA	NA	
Other	1	0.81 (0.61–1.09)	0		0	NA	NA		0	NA	NA	
**Magnesium intake type**												
Total	15			0.865	12			0.831	8			0.831
Total magnesium intake^c^	8	0.89 (0.82–0.96)	0		6	0.87 (0.80–0.94)	0		5	0.94 (0.79–1.12)	0	
Dietary magnesium intake	7	0.88 (0.81–0.96)	0.44		6	0.89 (0.77–1.03)	35.4		3	0.91 (0.70–1.18)	39.4	
**Difference between top and bottom intake (mg/day)^d^**												
Total	15			0.107	12			0.18	8			0.244
≥ 180	7	0.83 (0.76–0.91)	0		5	0.83 (0.76–0.91)	0		6	1.07 (0.83–1.37)	0	
< 180	8	0.93 (0.86–1.00)	0		7	0.92 (0.81–1.03)	26.2		2	0.89 (0.76–1.03)	0	
**Current CV events^e^**												
Total	15			0.074	12			0.393	8			NA
Yes	12	0.90 (0.85–0.96)	0		11	0.88 (0.81–0.96)	18.2		8	0.93 (0.82–1.06)	0	
Unknown	3	0.75 (0.63–0.90)	0		1	0.76 (0.57–1.01)	NA		0	NA	NA	
**Hypercholesterolemia^f^**												
Total	15			0.48	12			0.565	8			0.651
Yes	7	0.91 (0.83–0.99)	0		6	0.90 (0.80–1.01)	6.9		5	0.90 (0.76–1.08)	0	
Unknown	8	0.86 (0.79–0.95)	13.1		6	0.86 (0.77–0.97)	32.4		3	0.94 (0.72–1.22)	40.3	
**Current diabetes^g^**												
Total	15			0.039	12			0.159	8			NA
Yes	10	0.91 (0.82–0.97)	0		10	0.89 (0.82–0.97)	13.5		8	0.93 (0.82–1.06)	0	0
Unknown	5	0.75 (0.64–0.88)	0		2	0.72 (0.56–0.92)	0		0	NA	NA	NA

*BMI, body mass index; FFQ, food frequency questionnaire; SFFQ, semi-quantitative food frequency questionnaire; CV events, cardiovascular events; RR, relative risk; NA, not available*.

a *Several studies reported stroke outcome of male and female participants in different cohorts*.

b *Male and female participants were in the same cohort*.

c *Total magnesium intake (milligrams per day) included the total amount of magnesium from both food (diet) and supplements*.

d *Subtract the lowest category intake from the highest*.

e *Grouped by whether participants with or without CV events. CV events include coronary heart disease, heart attack, heart failure, atrial fibrillation, and self-reported heart disease etc.,hypertension (systolic blood pressure ≥ 140 mm Hg and/or diastolic blood pressure ≥ 90 mm Hg or on antihypertensive drugs use) in this part could be regarded as CV events. Stroke is not included*.

f *Grouped by whether participants with or without hypercholesterolemia. Hypercholesterolemia in this part means cholesterol concentration ≥ 240 mg/dL*.

g *Grouped by whether participants with or without diabetes*.

### Dose-Response Analysis

**Figure 4:**
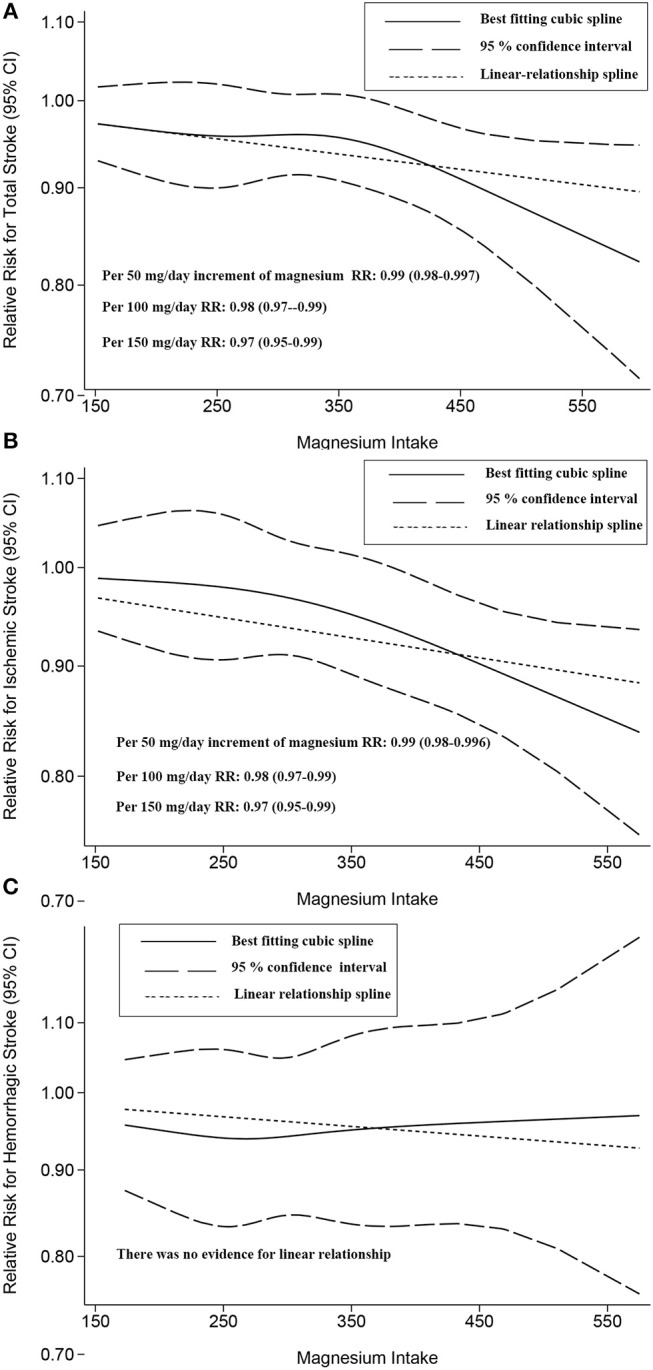
Two-stage dose-response effects on the relationships between magnesium intake and total stroke **(A)**; ischemic stroke **(B)**; hemorrhagic stroke **(C)**. The solid line represents non-linear estimates of the association between Magnesium Intake and the risk of expected outcomes; the dashed lines are the 95% confidence intervals (95% CIs); the dotted line represents the linear estimates of the associations between magnesium intake and the risk of expected outcomes. The vertical axis is the relative risk (RR) scale without logarithmic transformation.

Both linear and non-linear relationships were found for total stroke and for ischemic stroke (Figure 4). However, no dose-response relationship [non-linear (*P* = 0.345) or linear (*P* = 0.737)] was observed for hemorrhagic stroke (Figure 4). There was no evidence for a dose-response relationship between subarachnoid hemorrhage and intracerebral hemorrhage (Figure S12).

Related to a 100 mg/day increase in magnesium intake, for total stroke, the summary RR was 0.98 ([95% CI, 0.97–0.99]); for ischemic stroke, the RR was 0.98 ([95% CI, 0.97–0.99]). Overall, increased magnesium intake had a beneficial effect on these risk reductions.

### Publication Bias

No evidence of publication bias was observed for total stroke, ischemic stroke (Egger's test: *P* = 0.937) or hemorrhagic stroke (Egger's test: *P* = 0.809).

## Discussion

In 2010, the AHA Goals and Metrics Committee issued the 2020 Impact Goals to improve the cardiovascular health of all Americans by 20% while reducing the number of deaths due to cardiovascular disease by 20% (24); a crucial part of these goals is a healthy diet. According to a survey, dietary supplements are an ~$30 billion industry in the US, and some vitamins and nutritional supplements, such as folic acid, vitamin D, ω-3 fatty acids, and ω-3 polyunsaturated fatty acids, have been properly recommended for pregnant women, infants, children, and the elderly (25). The American Food and Nutrition Board's recommended dietary magnesium intake levels are 240 (9–13 y age)-420 mg/day (31–70 y age) for males and 240 (9–13 y age)-360 (14–18 y age), decreasing to 320 mg/day (31–70 y age), for females (26); an unfortunate problem is that people in developed countries seldom obtain adequate magnesium through their diets. As this comprehensive study showed a conclusive inverse association between magnesium intake and total stroke and ischemic stroke, there is a great necessity to correct magnesium intake deficiencies. The significance of magnesium intake (total and dietary) may be mentioned in guidelines for stroke prevention.

The 2015–2016 Dietary Guidelines for Americans suggest that all people follow a healthy eating pattern across the lifespan, and these dietary habits include consuming vegetables, fruits, whole grains, fat-free/low fat dairy, protein-rich foods, and oils (27). The 2014 Guidelines for the Primary Prevention of Stroke pinpointed that the Mediterranean and Dietary Approaches to Stop Hypertension (DASH) diets, which are rich in fruits and vegetables, would reduce stroke risk (3). Reducing the intake of sodium to a level below the current recommendations of 100 mmol per day will lower blood pressure; moreover, the DASH diet and making long-lasting dietary changes will bring individuals more long-term benefits (28). However, due to some confounders, there was no conclusive recommendation for magnesium intake (Table 2).

**Table 2:** The summary of relevant guidelines or advisories by influential colleges.

**Guidelines/advisories name**	**Year**	**Source/journal**	**Recommendations**
Diet and Lifestyle Recommendations Revision 2006	2006	AHA/Circulation	(1) Choose foods made with whole grains (such as whole wheat, oats/oatmeal, rye, barley, popcorn, brown rice, wild rice, buckwheat, triticale, bulgur (cracked wheat), millet, quinoa, and sorghum); (2) Increase intake of fruit and vegetables; (3) Available evidence is inadequate to recommend other dietary factors to reduce CVD risk.
Guidelines for the Primary Prevention of Stroke	2011	AHA, ASA/Stroke	(1) Reduce sodium and increase potassium intake; (2) A DASH-style diet and low fat diary is highly recommended; (3) Few randomized controlled trials with clinical outcomes have been conducted.
Guidelines for the Prevention of Stroke in Women	2014	AHA, ASA/Stroke	(1) Lifestyle factor such as a healthy diet reduces the risk of CVD and mortality; (2) Lifestyle interventions focusing on diet are recommended for primary stroke prevention among high risk individuals; (3) There are few published trials of lifestyle interventions for secondary stroke prevention.
Guidelines for the Primary Prevention of Stroke	2014	AHA, ASA/Stroke	(1) Reduce sodium and increase potassium intake; (2) A Mediterranean diet supplemented with nuts may lower the risk of stroke; (3) There is no conclusive evidence that vitamins or other nutrients (eg. magnesium) prevent stroke.
Dietary Guidelines for Americans 2015-2020 8th Edition	2015	Office of Disease Prevention and Health Promotion/NA	(1) Follow a healthy eating pattern across the lifespan; (2) Limit calories from added sugars and saturated fats and reduce sodium intake; (3) Nutritional needs should be met primarily from foods; (4) Role of magnesium is not well discussed.
Scientific Reports of the 2015 Dietary Guidelines Advisory Committee	2015	Department of Health and Human Services/NA	(1) Underconsumption of calcium, vitamin D, fiber, potassium, and iron (premenopausal women and adolescent females) is linked to health outcomes; (2) Nutrition and lifestyle interventions performed by multi-disciplinary teams should be emphasized; (3) Magnesium intake is always below national standards.
Medical Nutrition Education, Training, and Competences to Advance Guideline-Based Diet Counseling by Physicians	2018	AHA/Circulation	(1) A prudent dietary pattern can advance population-wide cardiovascular health;(2) Meta-analyses show fruits, vegetables, nuts and seeds, fish, total diary, cheese intake will significantly reduce risk of stroke; (3) Enhance physicians and individuals education and training in nutrition will reduce health and economic burden.

*AHA, American Heart Association; ASA, American Stroke Association; CVD, cardiovascular disease; DASH, dietary approaches to stop hypertension*.

In this study, we provided evidence for enhancing magnesium intake for stroke primary prevention. To the best of our knowledge, this is the first study to conduct a TSA. The conclusive results on total stroke and ischemic stroke required no further observational trials, thereby saving costs for public health administrations, especially on the present topic. Notably, we still warrant studies on hemorrhagic stroke and other randomized controlled trials (RCTs) on all discussed associations. In short, this topic involves a wide range of medical specialties: cardiology, neurology, neurosurgery, vascular surgery, intensive care, nutriology, and internal medicine. Future instructions may help to decrease the burden of stroke in large at-risk populations.

In terms of previous meta-analyses, based on 7 studies, Larsson et al. (17) found an 8% reduction in total stroke and a 9% reduction in ischemic stroke along with 100 mg/day increases in magnesium. However, the researchers failed to conduct a detailed subgroup analysis that included several confounders, such as dietary assessments and type of magnesium intake. The current study captured conclusive observational evidence and identified a robust inverse association between magnesium intake and total stroke. Compared to the group with the lowest intake of magnesium, Nie et al. (18) demonstrated an RR of 0.89 (95% CI: 0.82–0.97) for total stroke in the group with the highest intake. The essential subtypes of stroke were not available. Nie et al. (18) noted there was no significant inverse association in the male group or the European individuals group, a finding that was contradicted by the findings of our study. New evidence related to the two groups was entered into the present analysis, and the results significantly changed. A Mediterranean diet rich in magnesium is preferred by Western populations, including those in North America and Europe. The risk of coronary heart disease (CHD) was significantly reduced, particularly in males (16) compared to females. The reason females achieved more benefits in preventing total stroke is still unclear. This may be because females consumed more magnesium-rich diets in the primarily included studies, and studies have shown that individual blood pressure (BP) is well-controlled by magnesium in females (4, 7). When the follow-up period was prolonged, participants had a higher chance of turning to doctors for relief. Similar to stroke incidence, there were inverse associations between CHD and dietary magnesium or potassium intake. Previous cohort studies also illustrated that the intake of magnesium and calcium might lower CVD mortality (26), but the clear effects of magnesium and calcium on CVD requires further consideration. Fang et al. (19) investigated dietary magnesium intake in relation to type 2 diabetes and CVD risk and showed that stroke risk was reduced (RR, 0.88; 95% CI: 0.82–0.95) in a dose-dependent manner. The study only included dietary magnesium intake of the participants and ignored the subtypes of total stroke. From our perspective, current CVD status (excluding stroke) is a crucial confounder of the validated relationship and should have been addressed in the stratified analyses of the researchers. In the dose-response portion, when the highest or the lowest category was open-ended, Fang et al. (19) estimated the range of the category as the adjacent interval, which resulted in a summarized RR of 0.93 (95% CI: 0.89–0.87) per 100 mg/day increment. Current and previous meta-analyses did not support a beneficial role of magnesium in hemorrhagic stroke; however, patients with high serum magnesium levels might have fewer admission hematomas and better intracerebral hemorrhage prognoses (29).

The current study had several limitations. First, we did not ascertain the efficacy of magnesium supplements on stroke. Clinical trials on supplements have not demonstrated clear benefits for primary and secondary prevention of chronic diseases not related to nutrition deficiency, although they are highly taken by adults to maintain health (30). Second, the included NHS cohort and HPFS cohort have certain overlaps. Cohorts with varying follow-up periods with assessments from various investigators or at different time nodes may convey different characteristics of participants and results. We have shown that individuals with longer follow-ups (≥12 y) show larger benefits in the subgroup analyses. Third, most primary studies used FFQs/validated FFQs, which could not characterize all the nutrients and therefore did not clarify plausible associations. Fourth, observational evidence might only reach a conclusion but could not prove causality.

Researchers need to consider the impact of non-ignorable confounders in future guidelines or studies. Magnesium intake is associated with higher intakes of other potentially protective nutrients (e.g., potassium, folate, vitamin C), dietary fiber, and antioxidants. In addition, it may also be associated with other potential lifestyle factors (smoking) and other risk factors for stroke (hypertension). Thus, confounding cannot be excluded as a potential explanation for the observed inverse association of interest, and therefore, randomized controlled trials and other types of studies are needed to confirm the observational findings.

## Conclusion

Our definitive study with TSA showed magnesium intake has inverse association with total and ischemic stroke in a dose-response pattern. Additionally, it may also have a mild but not significant inverse association with hemorrhage stroke, subarachnoid hemorrhage and intracerebral hemorrhage. Magnesium consumption, herein, may be recommended as an optimization for stroke prevention or management. Most importantly, the cost-effective alternative debated by physicians, policy makers and legislators has a possibility to not only improve population-wide cardiovascular health but also guide policy decisions and initiate reform in global dietary healthcare. As for other chronic disease, whether such crucial population-based diet strategy may reduce public health and economic burden to an unprecedentedly low degree needs further exploration.

## Data Availability

No datasets were generated or analyzed for this study.

## Author Contributions

BZ had full access to all of the data in the manuscript and takes responsibility for the integrity of the data and the accuracy of the data analysis. BZ and WZ: drafting of the manuscript. BZ, WZ, YD, JinX, LH, DY, and JiaX: critical revision of the manuscript for important intellectual content. BZ: statistical analysis. WZ, JiaX, and YW: supervision. All authors: concept and design, acquisition, analysis, or interpretation of data.

### Conflict of Interest Statement

The authors have completed and submitted the ICMJE Form for Disclosure of Potential Conflicts of Interest.

## Supplementary Material

Figure S1 Flow chart of the literature search and screening process.

Figure S2 Trial Sequential Analysis (TSA) of total stroke with the included studies indicated.

Figure S3 Forest Plots of the Risk of Ischemic Stroke for Magnesium Intake **(A)** and for <50 mg/day **(B)**, ≥50 and <100 mg/day **(C)**, ≥100 and <150 mg/day **(D)**, and ≥150 mg/day magnesium increments **(E)**.

Figure S4 Trial Sequential Analysis (TSA) for ischemic stroke with included studies indicated.

Figure S5 Forest plots of the risk of hemorrhagic stroke for magnesium intake **(A)** and for <50 mg/day **(B)**, ≥50 and <100 mg/day **(C)**, ≥100 and <150 mg/day **(D)**, and ≥150 mg/day magnesium increments **(E)**.

Figure S6 Trial sequential analysis of hemorrhagic stroke comparing the highest magnesium intake category to the lowest.

Figure S7 Trial Sequential Analysis (TSA) for hemorrhagic stroke with the included studies indicated.

Figure S8 Forest plots of the risk of subarachnoid hemorrhage for magnesium intake **(A)** and for <50 mg/day **(B)**, ≥50 and <100 mg/day **(C)**, ≥100 and <150 mg/day **(D)** and ≥150 mg/day magnesium ranges **(E)**.

Figure S9 Trial Sequential Analysis (TSA) of subarachnoid hemorrhage **(A)**, and the TSA of subarachnoid hemorrhage with the included studies indicated **(B)**.

Figure S10 Forest plots of the risk of intracerebral hemorrhage for magnesium intake **(A)** and for <50 mg/day **(B)**, ≥50 and <100 mg/day **(C)**, ≥100 and <150 mg/day **(D)**, and ≥150 mg/day magnesium ranges **(E)**.

Figure S11 Trial Sequential Analysis (TSA) of intracerebral hemorrhage **(A)**, and the TSA of intracerebral hemorrhage with the included studies indicated **(B)**.

Figure S12 Two-stage dose-response effects on the relationships between magnesium intake and subarachnoid hemorrhage **(A)** and intracerebral hemorrhage **(B)**.

Table S1 PRISMA 2009 checklist.

Table S2 Characteristics of included eligible studies.

Table S3 Methodological quality assessments of the studies included with the newcastle-ottawa scales.

## Abbreviations

RR: Relative risk
CI: Confidence intervals
BMI: Body mass index
PRISMA: Preferred Reporting Items for Systematic Reviews and Meta-Analyses
NOS: Newcastle-Ottawa Scale
CV: Cardiovascular
RCTs: Randomized controlled trials
DALYs: Disability-adjusted life-years
ASA: American Stroke Association
CHD: Coronary heart disease
BP: Blood pressure.
