# Supplementary figures and images for: The Effect of Magnesium Intake on Stroke Incidence: A Systematic Review and Meta-Analysis With Trial Sequential Analysis
Source: Front Neurol. 2019 Aug 7;10:852. doi: 10.3389/fneur.2019.00852 (PMC6692462; doi:10.3389/fneur.2019.00852)

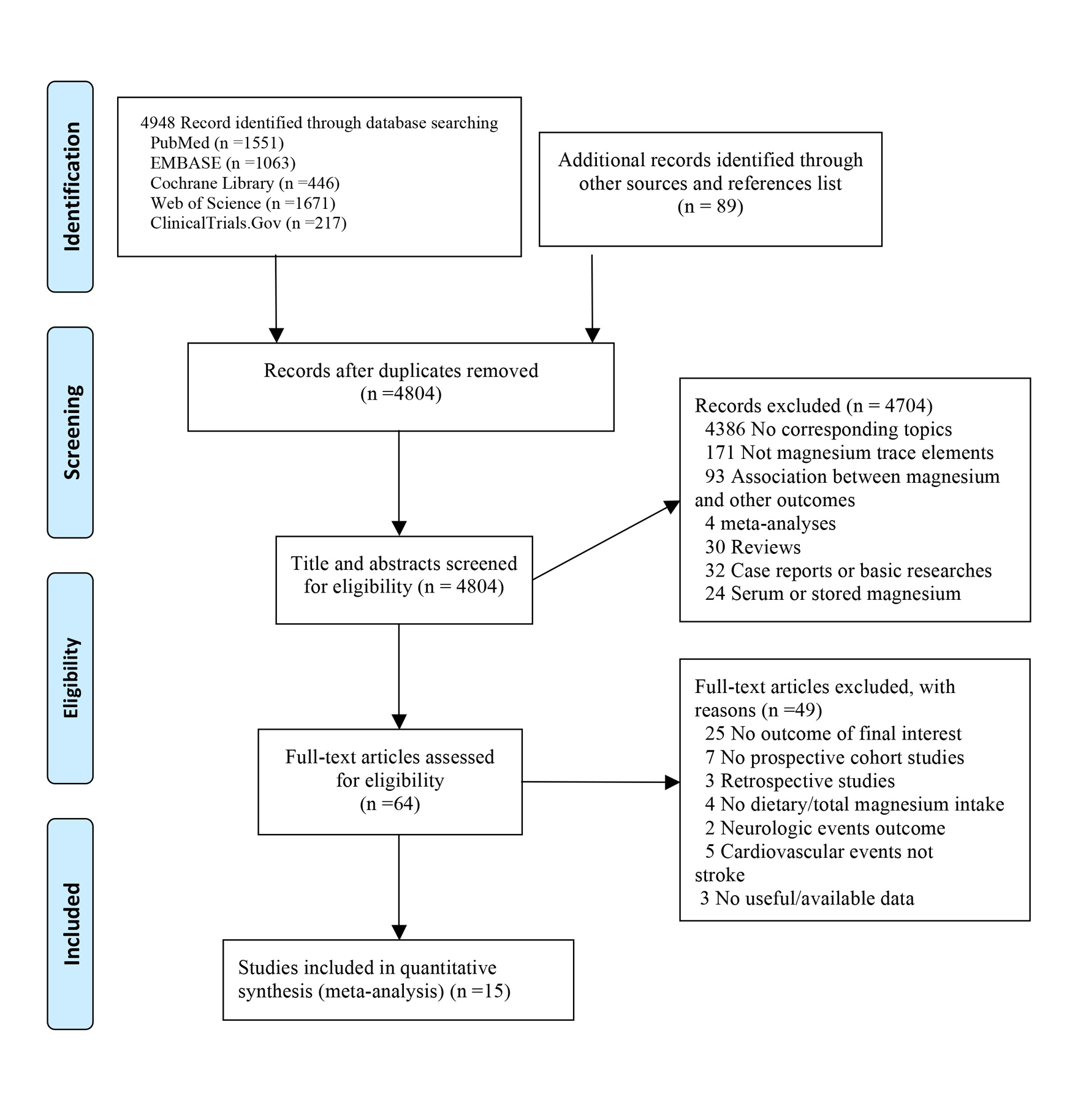

Supplement: Figure S1 — Flow chart of the literature search and screening process. [file Image_1.TIF]

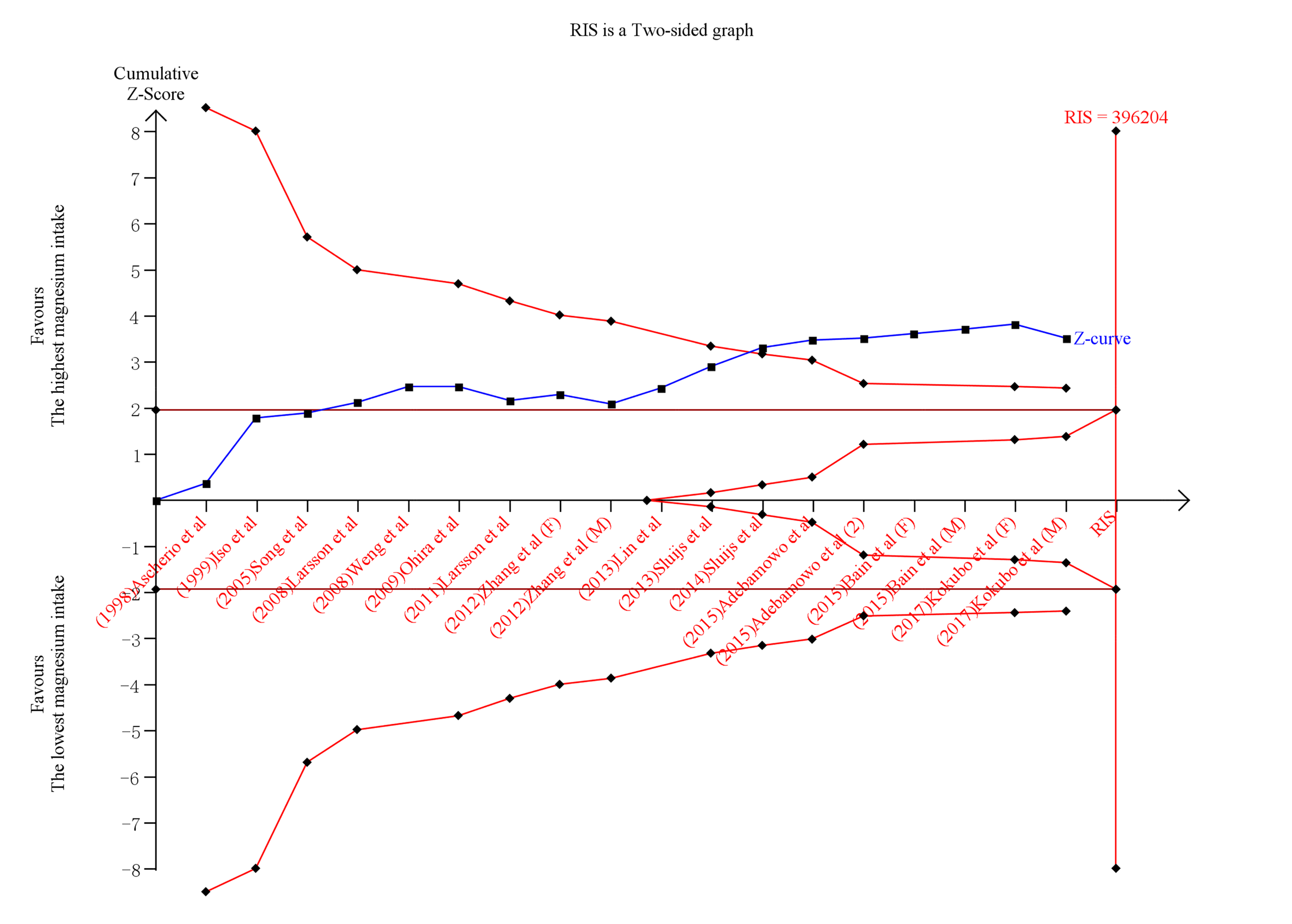

Supplement: Figure S2 — Trial Sequential Analysis (TSA) of total stroke with the included studies indicated. [file Image_2.TIF]

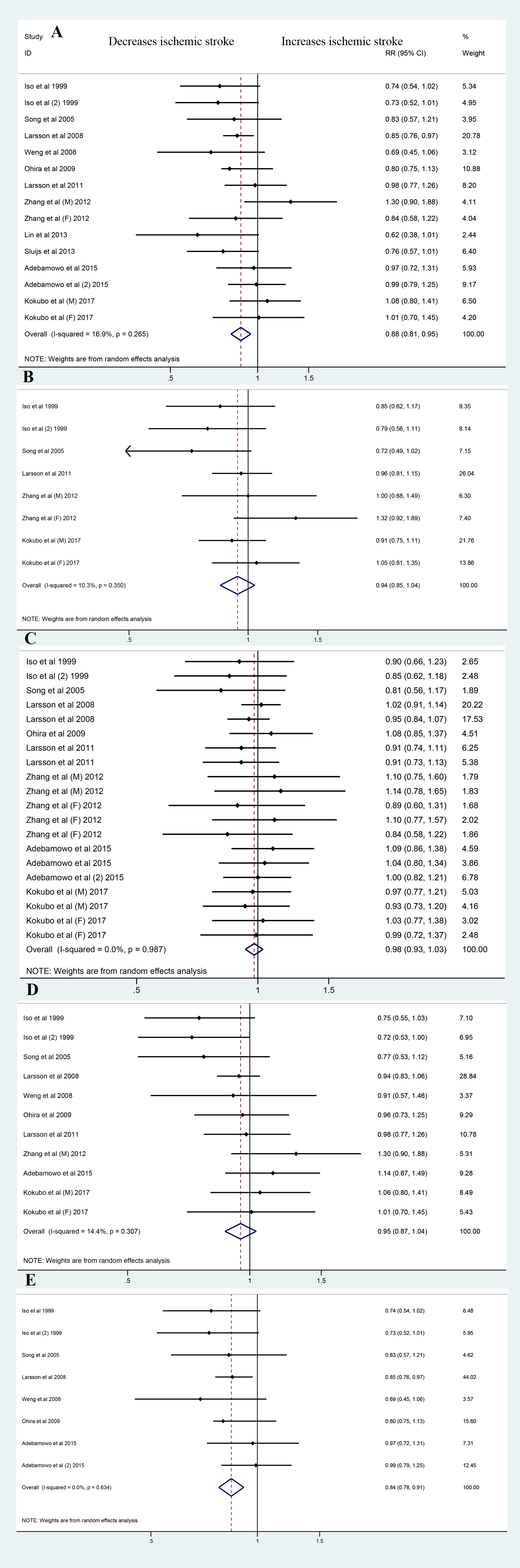

Supplement: Figure S3 — Forest Plots of the Risk of Ischemic Stroke for Magnesium Intake (A) and for <50 mg/day (B), ≥50 and <100 mg/day (C), ≥100 and <150 mg/day (D), and ≥150 mg/day magnesium increments (E). [file Image_3.TIF]

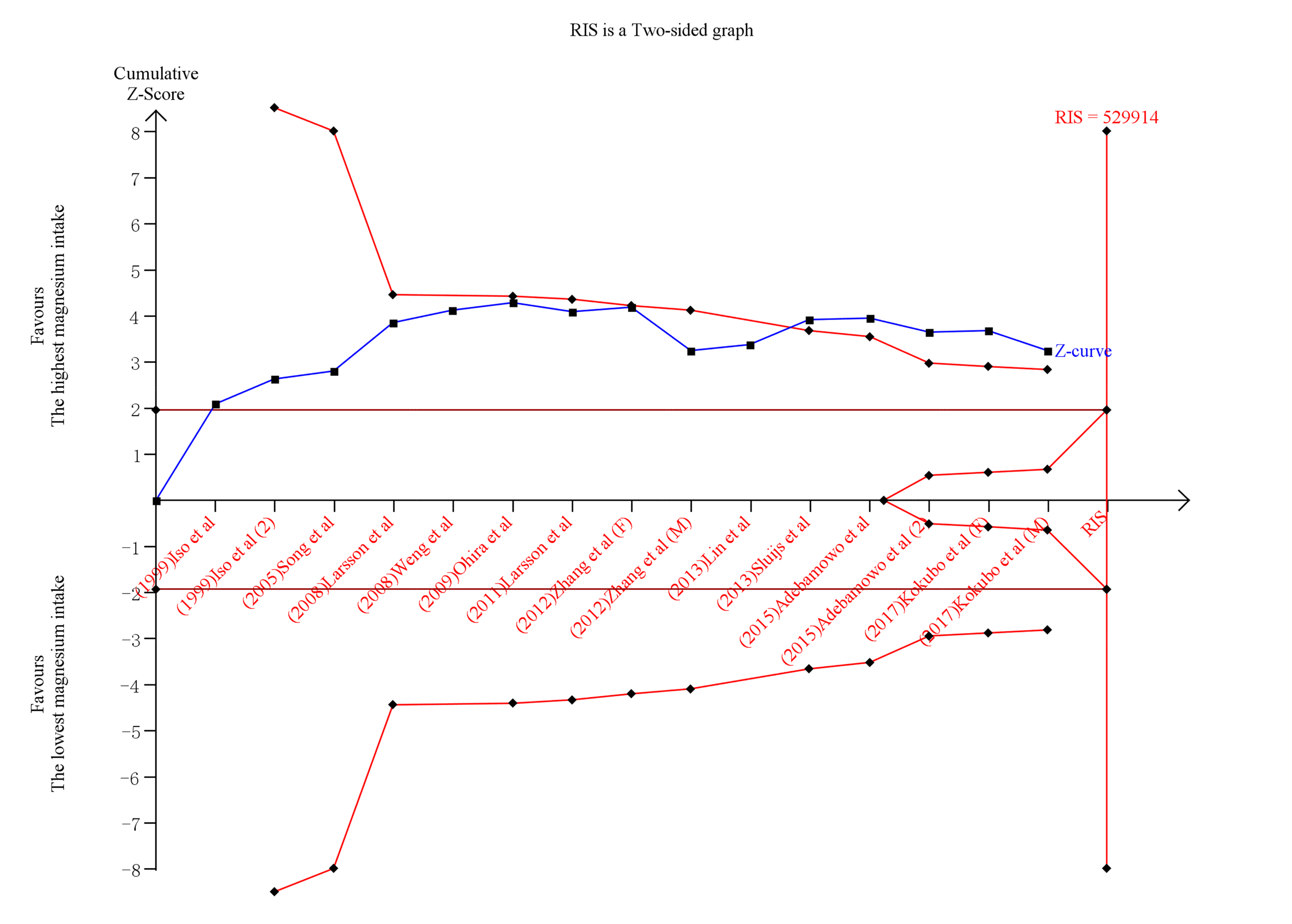

Supplement: Figure S4 — Trial Sequential Analysis (TSA) for ischemic stroke with included studies indicated. [file Image_4.TIF]

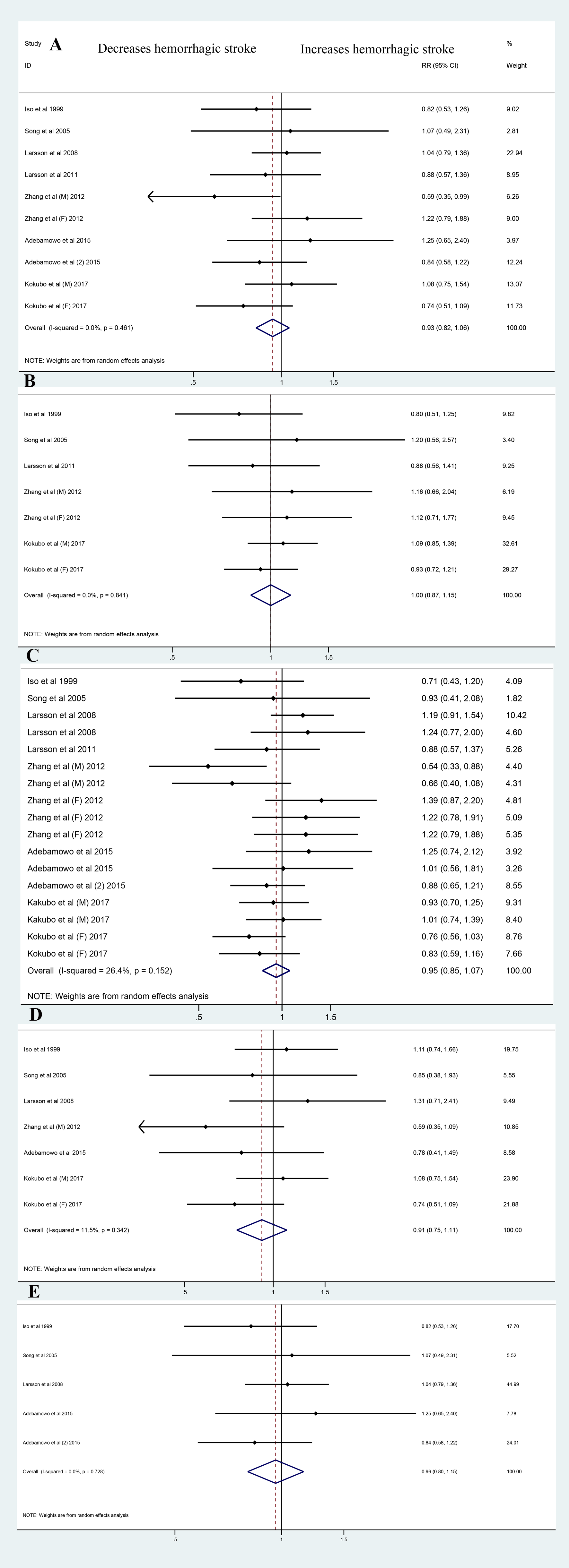

Supplement: Figure S5 — Forest plots of the risk of hemorrhagic stroke for magnesium intake (A) and for <50 mg/day (B), ≥50 and <100 mg/day (C), ≥100 and <150 mg/day (D), and ≥150 mg/day magnesium increments (E). [file Image_5.TIF]

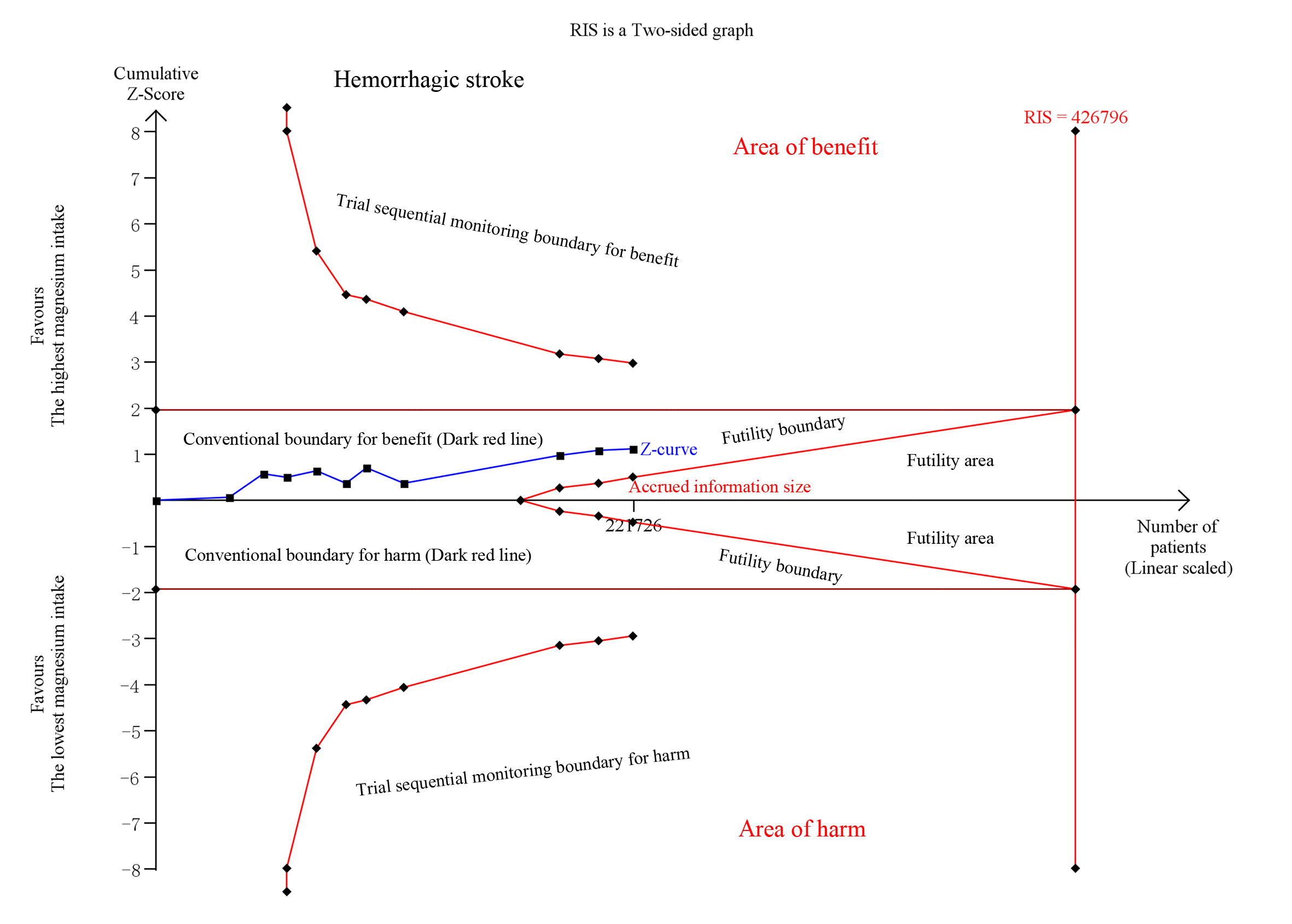

Supplement: Figure S6 — Trial sequential analysis of hemorrhagic stroke comparing the highest magnesium intake category to the lowest. [file Image_6.TIF]

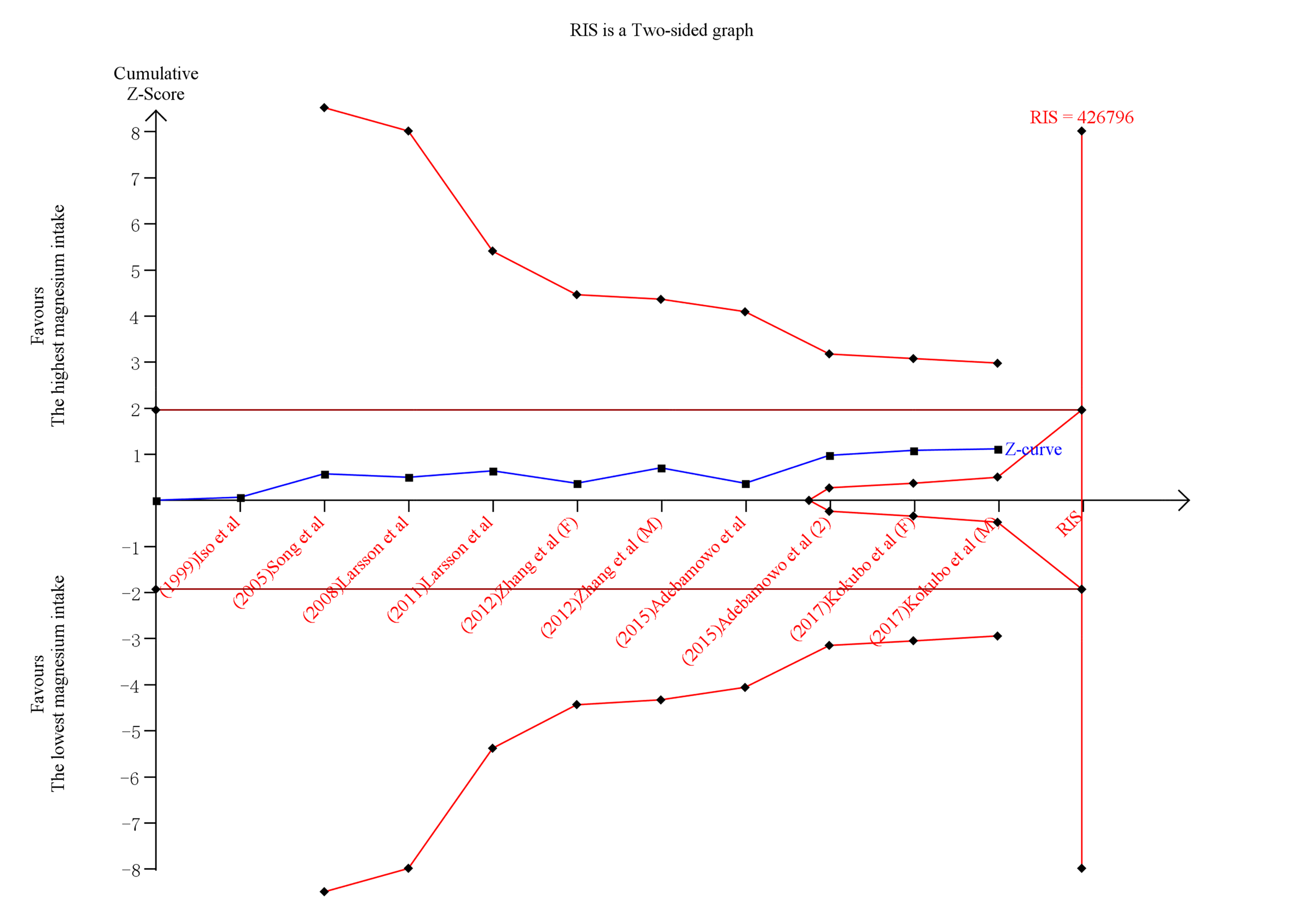

Supplement: Figure S7 — Trial Sequential Analysis (TSA) for hemorrhagic stroke with the included studies indicated. [file Image_7.TIF]

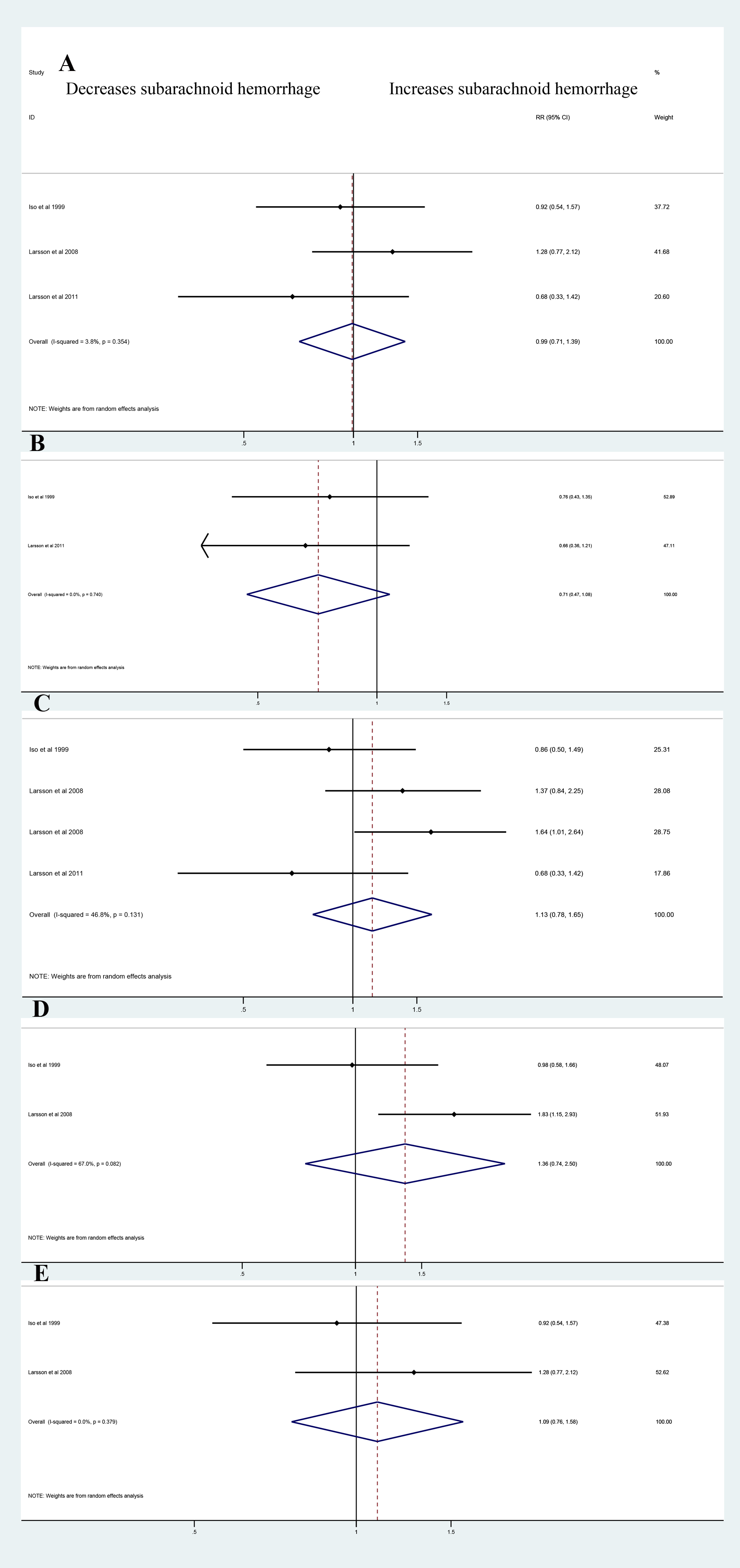

Supplement: Figure S8 — Forest plots of the risk of subarachnoid hemorrhage for magnesium intake (A) and for <50 mg/day (B), ≥50 and <100 mg/day (C), ≥100 and <150 mg/day (D) and ≥150 mg/day magnesium ranges (E). [file Image_8.TIF]

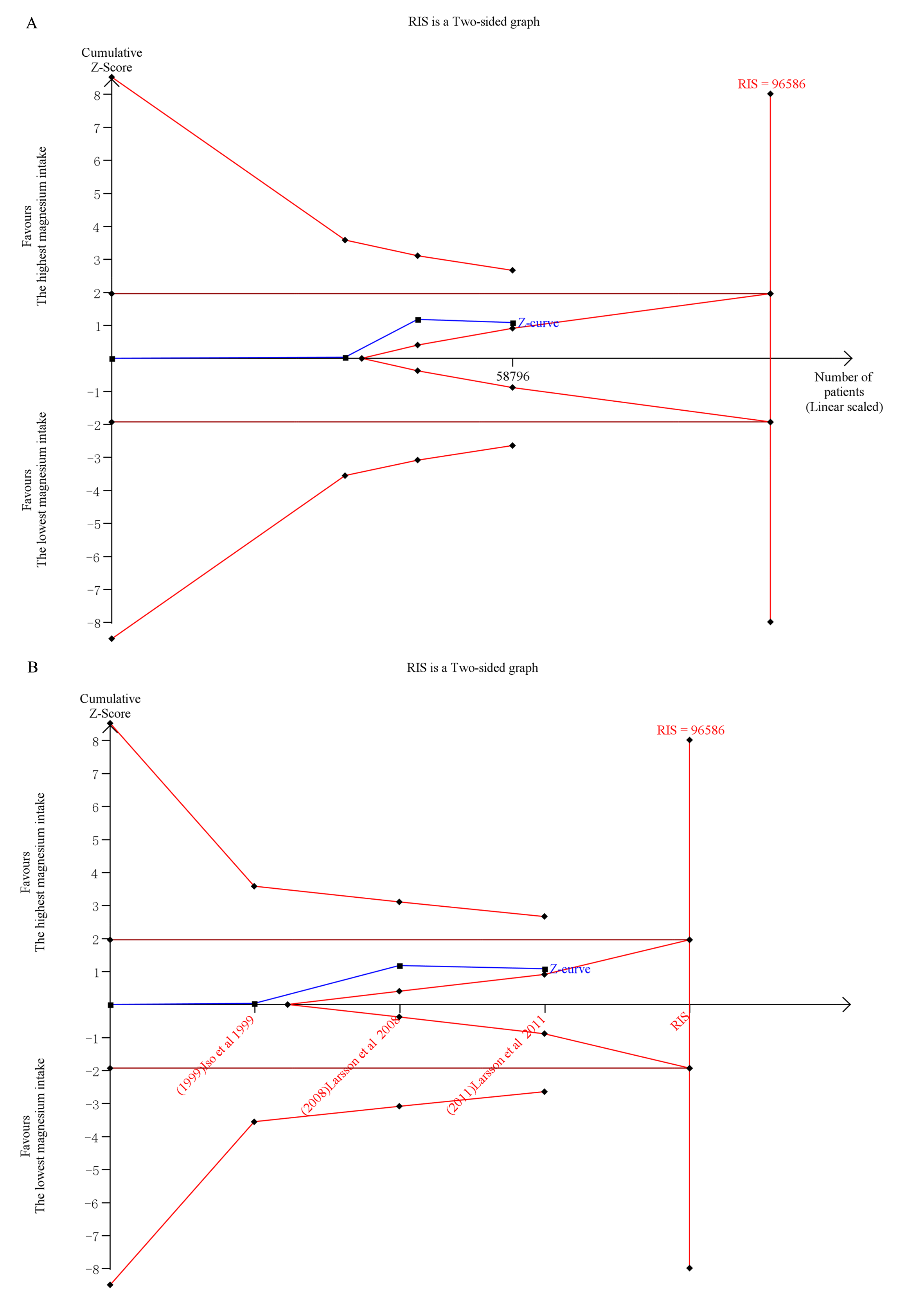

Supplement: Figure S9 — Trial Sequential Analysis (TSA) of subarachnoid hemorrhage (A), and the TSA of subarachnoid hemorrhage with the included studies indicated (B). [file Image_9.TIF]

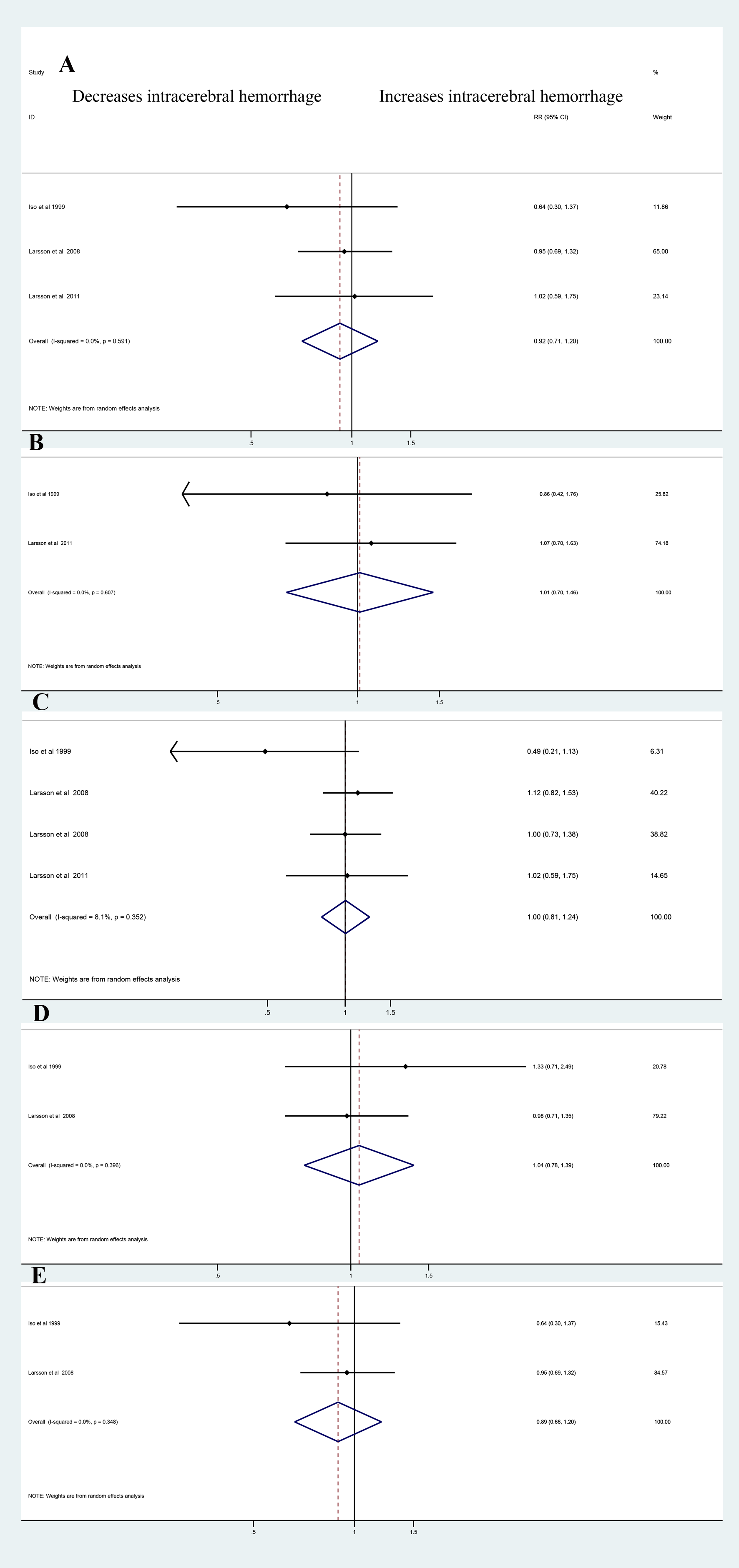

Supplement: Figure S10 — Forest plots of the risk of intracerebral hemorrhage for magnesium intake (A) and for <50 mg/day (B), ≥50 and <100 mg/day (C), ≥100 and <150 mg/day (D), and ≥150 mg/day magnesium ranges (E). [file Image_10.TIF]

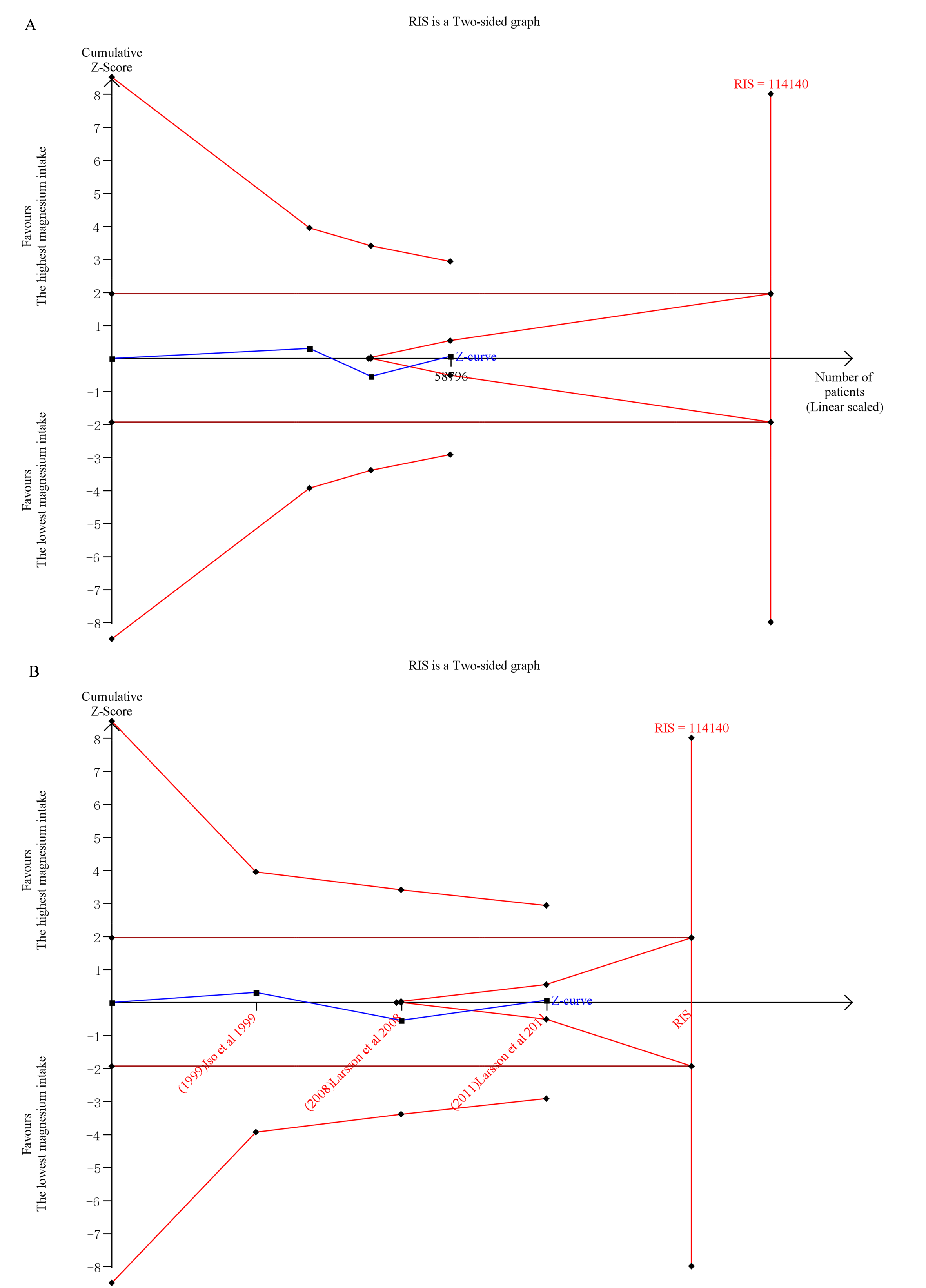

Supplement: Figure S11 — Trial Sequential Analysis (TSA) of intracerebral hemorrhage (A), and the TSA of intracerebral hemorrhage with the included studies indicated (B). [file Image_11.TIF]

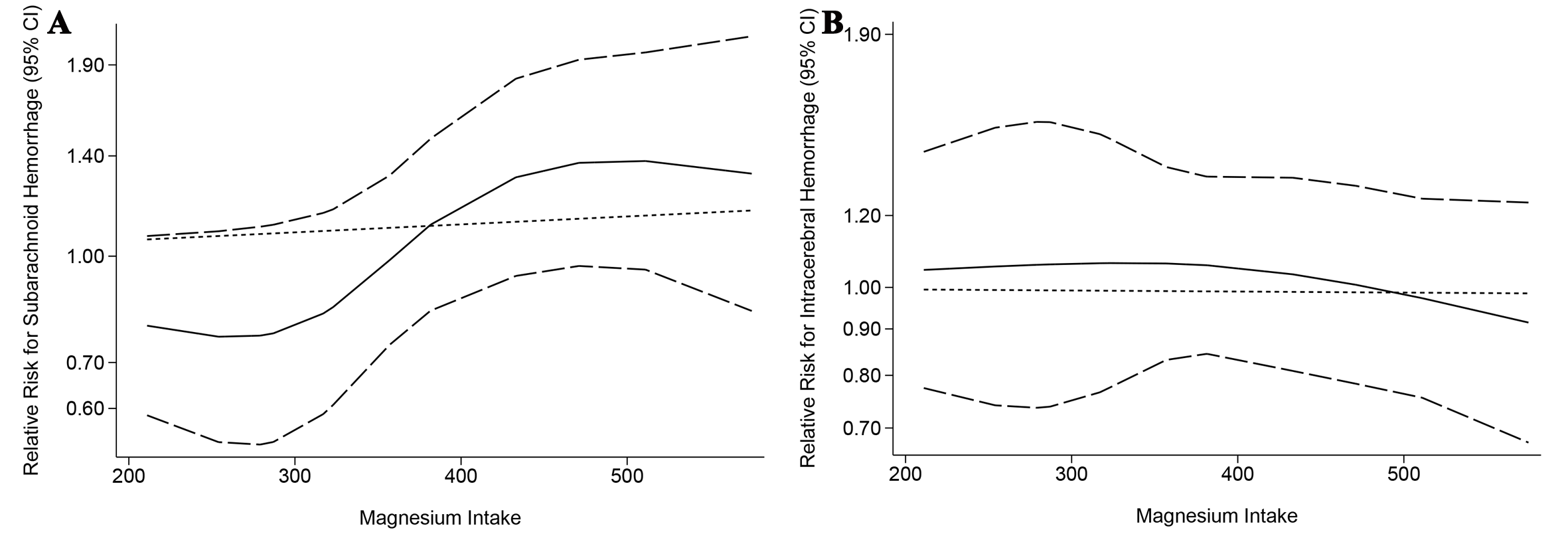

Supplement: Figure S12 — Two-stage dose-response effects on the relationships between magnesium intake and subarachnoid hemorrhage (A) and intracerebral hemorrhage (B). [file Image_12.TIF]
