# Supplementary material for: The Effect of Magnesium Intake on Stroke Incidence: A Systematic Review and Meta-Analysis With Trial Sequential Analysis
Source: Front Neurol. 2019 Aug 7;10:852. doi: 10.3389/fneur.2019.00852 (PMC6692462; doi:10.3389/fneur.2019.00852)
Supplement: Table S2 — Characteristics of included eligible studies. [file Table_2.DOCX]

**Table S2. Characteristics of Included Eligible Studies**

| Source | Period/Duration (y) | Population | BMI | Dietary Assessment | Case Ascertainment | Case (Cohort size) | Magnesium intake (mg/day) highest VS. the lowest [Adjusted RR (95% CI)] | Adjustment for potential confounders^a^ |
| --- | --- | --- | --- | --- | --- | --- | --- | --- |
| Ascherio et al,[^12^](#_ENREF_12) 1998 (America) | 1986-1994/8 | M; 40-75 y | NA | validated FFQ | self-reported questionnaire | 328 cases of total stroke (43738) | 425 VS. 243 (0.92 (0.58-1.46)) | Age, smoking, profession, histories of hypertension and hypercholesterolemia, family history of MI, BMI, physical activity, and intakes of alcohol, dietary fiber, potassium, and total energy |
| Iso et al,^13^ 1999 (America) | 1980-1994/14 | F; 34-59 y | 22.7 | FFQ | self-reported questionnaire | 690 cases of total stroke (85764) | 381 VS. 211 (0.80 (0.63-1.01)) | Age, smoking, menopausal status, postmenopausal hormone use, BMI, exercise, histories of diabetes and high cholesterol, aspirin use, multivitamin use, vitamin E use, alcohol, omega-3 fatty acids, and calcium |
| Song et al,^14^ 2005 (America) | 1993-2003/10 | F; 39-89 y | 26 | FFQ | follow-up examination | 368 cases of total stroke (39876) | 433 VS. 255 (0.90 (0.65-1.26)) | Age, randomized treatment assignment, smoking, BMI, exercise, postmenopausal hormone use, multivitamin use, histories of diabetes, hypertension, and hypercholesterolemia, family history of MI, intake of alcohol, and total energy |
| Larsson et al,^15^ 2008 (Sweden) | 1985-2004/13.6 | M; 50-69 y | 26.4 | validated FFQ | follow-up examination | 3370 cases of total stroke (26556) | 575 VS. 382 (0.91 (0.77-1.07)) | Age, supplementation group, cigarettes smoked daily, BMI, physical activity, SBP, DBP, serum total and HDL cholesterol, histories of diabetes and ischemic heart disease, alcohol, energy intake |
| Weng et al,^16^ 2008 (Taipei) | 1989-2002/10.6 | M/F; ≥40 y | 24.5 | validated FFQ | Self-reported and cross-checked questionnaire | 132 cases of ischemic stroke (1772) | 423 VS. 162 (0.69 (0.45-1.06)) | Age, sex, age*sex, smoking, sex*smoking, area, central obesity, BMI, diabetes, physical activity, hypertension, use of antihypertensive drugs, self reported heart disease, hypercholesterolemia, hypertriglyceridemia, fibrinogen, apolipoprotein B, plasminogen, alcohol |
| Ohira et al,^17^ 2009 (America) | 1987-2004/15 | M/F; 45-64 y | 27.4 | validated FFQ | follow-up examination | 577 cases of ischemic stroke (14221) | 362 VS. 152 (0.80 (0.75-1.13)) | Age, sex, location, education, smoking, BMI, diabetes, SBP, use of antihypertensive medication, LDL, HDL, fibrinogen, von Willebrand factor, energy intake |
| Larsson et al,^18^ 2011 (Sweden) | 1998-2008/10.4 | F; 49-83 y | 25 | validated FFQ | follow-up examination | 1680 cases of total stroke (34670) | 373 VS. 297 (1.02 (0.82-1.27)) | Age, education, smoking, BMI, physical activity, history of diabetes, history of hypertension, aspirin use, family history of myocardial infarction, and alcohol, protein, cholesterol, total fiber, folate, and energy intake |
| Zhang et al,^19^ 2012 (Japan) | 1988-2006/14.7 | M; 40-79 y | 22.7 | validated FFQ | follow-up examination | 634 cases of total stroke (23083) | 294 VS. 173 (1.03 (0.79-1.35)) | Age, BMI, smoking, ethanol intake, history of hypertension, history of diabetes, physical activity, education, mental stress, menopausal status, hormone therapy |
|  |  | F; 40-79 y | 22.9 |  |  | 620 cases of total stroke (35533) | 274 VS. 175 (0.90 (0.69-1.16)) |  |
| Lin et al,^20^ 2013 (Taipei) | 1989-2002/13 | M/F; ≥ 18 y | 23.3 | validated FFQ | follow-up examination and self-reported questionnaire | 123 cases of total stroke (2061) | 378 VS. 210 (0.62 (0.40-0.97)) | Age, urinary sodium/creatinine, alcohol, physical activity, SBP, DBP, anti-hypertension medication |
| Sluijs et al,^21^ 2013 (Netherland) | NA/12 | M/F; 21-70 y | NA | FFQ | NA | 361 cases of ischemic stroke (36359) | 435 VS. 253 (0.76 (0.57-1.01)) | Age, sex, BMI, education, physical activity, smoking, alcohol |
| Sluijs et al,^22^ 2014 (Netherland) | NA/12 | M/F; 21-70 y | NA | FFQ | follow-up examination | 631 cases of total stroke (36094) | 597 VS. 190 (0.64 (0.44-0.94)) | Age, sex, BMI, education, physical activity, smoking, alcohol, energy intake, calcium, potassium |
| Adebamowo et al,^23^ 2015 (America) | 1986-2010/24 | M; 40-75 y | 25.4 | validated FFQ | self-reported questionnaire | 1547 cases of total stroke (42669) | 467 VS. 267 (0.89 (0.71-1.11)) | Age, calendar year, total calories, smoking, BMI, parental history of heart disease, alcohol, physical activity, aspirin, multivitamin, history of hypertension, hypercholesterolemia, diabetes at baseline, and thiazide, potassium, calcium, |
| Adebamowo et al (2),^24^ 2015 (America) | 1976-2006/30 | F; 30-55 y | 26.4 | validated FFQ | self-reported questionnaire | 3237 cases of total stroke (86149) | 411 VS. 233 (0.93 (0.79-1.08)) | Age, calendar year, total calories , BMI, family history of heart disease, alcohol, physical activity, smoking, menopausal status, hormone therapy, oral contraceptive use, aspirin , multivitamin, history of hypertension, hypercholesterolemia, diabetes, thiazide use, potassium, calcium |
|  | 1989-2011/22 | F; 25-42 y | 25.7 |  |  | 543 cases of total stroke (94715) |  |  |
| Bain et al,^25^ 2015 (Britain) | 2002-2008/5.8 | M; 40-75 y | 26.5 | 7-day diary recall | follow-up examination | 364 cases of total stroke (2000) | 456 VS. 266 (0.81 (0.53-1.22)) | Age, BMI, education, physical activity, smoking, alcohol, serum total cholesterol, history of MI, history of diabetes, family history of stroke, family history of MI, SBP, DBP, aspirin, antihypertensive medication |
|  |  | F; 40-75 y | 26.2 |  |  | 511 cases of total stroke (2445) | 374 VS. 456 (0.82 (0.54-1.24)) |  |
| Kokubo et al,^26^ 2017 (Japan)^b^ | 1990-2009/15.2 | M; 40-69 y | 23.6 | FFQ | follow-up examination | 2576 cases of total stroke (39505) | 348 VS. 213 (1.07 (0.86-1.33)) | Age, smoking, alcohol, BMI, history of diabetes, antihypertensive medication, antilipidemic medication users, physical activity, dietary intake of fruits, vegetables, fish, dietary energy, location, minerals intake |
|  | 1993-2010/15.2 | F; 40-69 y | 23.6 |  |  | 1846 cases of total stroke (45788) | 333 VS. 213 (0.88 (0.67-1.14)) |  |
| Abbreviations: FFQ, food-frequency questionnaire; SFFQ, semi-quantitative food-frequency questionnaire; BMI, body mass index; MI, myocardial infarction; SBP, systolic blood pressure; DBP, diastolic blood pressure; HDL, high density lipoprotein; GI, glycemic index; NA, not available.  ^a^, in fully-adjusted model;  ^b^, the dose of magnesium intake that is not available in this study is retrieved from the same cohort reported in former publication. | | | | | | | | |
|  |  |  |  |  |  |  |  |  |
|  |  |  |  |  |  |  |  |  |
|  |  |  |  |  |  |  |  |  |
