# Supplementary material for: The Effect of Magnesium Intake on Stroke Incidence: A Systematic Review and Meta-Analysis With Trial Sequential Analysis
Source: Front Neurol. 2019 Aug 7;10:852. doi: 10.3389/fneur.2019.00852 (PMC6692462; doi:10.3389/fneur.2019.00852)
Supplement: Table S3 — Methodological quality assessments of the studies included with the newcastle-ottawa scales. [file Table_3.DOCX]

**Table S3. Methodological Quality Assessments of Included Studies with the Newcastle-Ottwa Scales**

|  | Study | Selection | | | | Comparability | Outcome | | | Total score |
| --- | --- | --- | --- | --- | --- | --- | --- | --- | --- | --- |
|  |  | Exposed cohort | Nonexposed cohort | Ascertainment of exposure | Outcome of interest |  | Assessment of outcome | Length of follow-up | Adequacy of follow-up |  |
| 1998 | Ascherio et al,^12^ | * | * | * | * | ** | * | * | * | 9 |
| 1999 | Iso et al,^13^ | * | * | * | * | ** | * | * | * | 9 |
| 2005 | Song et al,^14^ | * | * | * | * | ** | * | * | * | 9 |
| 2008 | Larsson et al,^15^ | * | * | * | * | ** | * | * | * | 9 |
| 2008 | Weng et al,^16^ | * | * | * | * | ** | * | * | * | 9 |
| 2009 | Ohira et al,^17^ | * | * | * | * | ** | * | * | * | 9 |
| 2011 | Larsson et al,^18^ | * | * | * | * | ** | * | * | * | 9 |
| 2012 | Zhang et al,^19^ | * | * | * | * | ** | * | * | * | 9 |
| 2013 | Lin et al,^20^ | * | * | * | * | ** | * | * | * | 9 |
| 2013 | Sluijs et al,^21^ | * | * | * | * | ** |  | * | * | 8 |
| 2014 | Sluijs et al,^22^ | * | * | * | * | ** | * | * | * | 9 |
| 2015 | Adebamowo et al,^23^ | * | * | * | * | ** | * | * | * | 9 |
| 2015 | Adebamowo et al (2),^24^ | * | * | * | * | ** | * | * | * | 9 |
| 2015 | Bain et al,^25^ | * | * | * | * | ** | * | * | * | 9 |
| 2017 | Kokubo et al,^26^ | * | * | * | * | ** | * | * | * | 9 |
